# P2X7R-mediated autophagic impairment contributes to central sensitization in a chronic migraine model with recurrent nitroglycerin stimulation in mice

**DOI:** 10.1186/s12974-020-02056-0

**Published:** 2021-01-05

**Authors:** Li Jiang, Yixin Zhang, Feng Jing, Ting Long, Guangcheng Qin, Dunke Zhang, Lixue Chen, Jiying Zhou

**Affiliations:** 1 Department of Neurology, The First Affiliated Hospital of Chongqing Medical University, 1st You Yi Road, Yuzhong District, Chongqing, 400016 China; 2 Department of Neurology, Chongqing General Hospital, Chongqing, China; 3 Department of Neurology, The Affiliated Hospital of Southwest Medical University, Luzhou, China; 4 Laboratory Research Center, The First Affiliated Hospital of Chongqing Medical University, Chongqing, China

**Keywords:** P2X7R, Autophagy, Microglia, Inflammation, Central sensitization, Chronic migraine

## Abstract

**Background:**

Central sensitization is an important pathophysiological mechanism of chronic migraine (CM). According to our previous studies, microglial activation and subsequent inflammation in the trigeminal nucleus caudalis (TNC) contribute to the central sensitization. The P2X7 receptor (P2X7R) is a purinergic receptor expressed in microglia and participates in central sensitization in chronic pain, but its role in CM is unclear. Numerous studies have shown that P2X7R regulates the level of autophagy and that autophagy affects the microglial activation and inflammation. Recently, autophagy has been shown to be involved in neuropathic pain, but there is no information about autophagy in CM. Therefore, the current study investigated the role of P2X7R in CM and its underlying mechanism, focusing on autophagy regulation.

**Methods:**

The CM model was established by repeated intraperitoneal injection of nitroglycerin (NTG) in mice. A Von Frey filament and radiant heat were used to assess the mechanical and thermal hypersensitivity. Western blotting and immunofluorescence assays were performed to detect the expression of P2X7R, autophagy-related proteins, and the cellular localization of P2X7R. To determine the role of P2X7R and autophagy in CM, we detected the effects of the autophagy inducer, rapamycin (RAPA) and P2X7R antagonist, Brilliant Blue G (BBG), on pain behavior and the expression of calcitonin gene-related peptide (CGRP) and c-fos. In addition, the effect of RAPA and BBG on microglial activation and subsequent inflammation were investigated.

**Results:**

The expression of P2X7R was increased and was mainly colocalized with microglia in the TNC following recurrent NTG administration. The autophagic flux was blocked in CM, which was characterized by upregulated LC3-II, and accumulated autophagy substrate protein, p62. RAPA significantly improved the basal rather than acute hyperalgesia. BBG alleviated both basal and acute hyperalgesia. BBG activated the level of autophagic flux. RAPA and BBG inhibited the activation of microglia, limited the inflammatory response, and reduced the expression of CGRP and c-fos.

**Conclusions:**

Our results demonstrate the dysfunction of the autophagic process in CM. Activated autophagy may have a preventive effect on migraine chronification. P2X7R contributes to central sensitization through mediating autophagy regulation and might become a potential target for CM.

## Background

Chronic migraine (CM) is the most common disorder of chronic daily headache, which is manifested as headache for at least 15 days a month [1]. Due to the frequent and severe headache attacks, CM has a high disability rate and great disease burden. At present, the management of CM is still a major challenge for neurologists due to the limited treatment choices, inadequate evidence of prophylactics, and poor treatment response [2]. Improved knowledge of the precise pathogenesis might benefit the development of new therapeutic targets for CM.

It is widely accepted that central sensitization, which is characterized by increased excitability of neurons in the central trigeminal pathway, plays a critical role in the development and progression of CM [3, 4]. Previous research associated with the mechanism underlying central sensitization has mainly focused on neurons, while recent studies confirm that microglia in the central nervous system (CNS) are also involved in this process [5, 6]. Our previous works have shown that the activated microglia in the trigeminal nucleus caudalis (TNC) area contribute to central sensitization in the CM by releasing inflammatory mediators and neurotrophic factors and subsequent microglia-neuron interactions [7–10].

The theory that purinergic signaling participates in the pathophysiology of migraine was proposed by Geoffrey Burnstock in the 1980 [11]. The purinergic receptors, P2X4R and P2Y12R, were confirmed to contribute to the pathogenesis of CM according to our previous works [7, 9]. P2X7R, an ionotropic purinergic receptor that is widely expressed in the microgli a[12], has been reported to be involved in the cancer pain, neuropathic pain, and inflammatory pain by mediating the microglial activation and the inflammatory response [13–15]. In the acute migraine model established by single nitroglycerin (NTG) injection, P2X7R inhibition attenuated NTG-induced thermal hyperalgesia and c-fos expression in the TNC area, suggesting that P2X7R might participate in the pathophysiology of migraine [16]. However, the role and specific mechanism of P2X7R in the chronic process of CM have not been explored to date.

Autophagy is an ubiquitous and conserved cellular process in which damaged organelles and abnormal proteins are delivered to lysosomes for degradation [17]. This process is essential to maintain the homeostasis of the cellular environment and achieve the requirements of cellular metabolism as well as organelle turnover [18]. Autophagy is regulated by immune signals [19]. Numerous studies have indicated the critical role of P2X7R in autophagy regulation [20–23]. The P2X7R-mediated autophagy modulation has been confirmed to participate in the pathophysiological process of amyotrophic lateral sclerosis (ALS) [22], Duchenne muscular dystrophy [23], and status epilepticus [21]. Meanwhile, extensive evidence has revealed the interaction between autophagy and inflammatory processes. Autophagy can regulate the activation of microglia and subsequent inflammatory response [24, 25]. Dysfunctional autophagy promotes microglial activation through regulating the production of IL1β and IL18 via NLRP3 degradation, and it contributes to neuroinflammation in Alzheimer’s disease (AD) [26]. We have previously confirmed the pivotal role of NLRP3 inflammasome activation in the central sensitization in CM [10]. In addition, altered expression of the autophagy marker in neuropathic pain (Nep) indicates that autophagy is involved in the modulation of pain processing [27, 28]. However, the effect of autophagy on migraine has not been identified. The relationship between autophagy and P2X7R in CM has not been previously investigated.

Therefore, in this study, we evaluated the level of autophagy in CM and identified its effect on pain relief. Then, we investigated the role of P2X7R in CM and explored the underlying molecular mechanisms, mainly focusing on autophagy regulation. Our data revealed the blockage of autophagy in NTG-induced CM and the protective effect of autophagy on CM. Microglial P2X7R was upregulated following NTG injection and contributed to central sensitization by regulating the activation of microglia and inflammation via autophagy modulation.

## Methods

### Animals

**Fig. 1:**
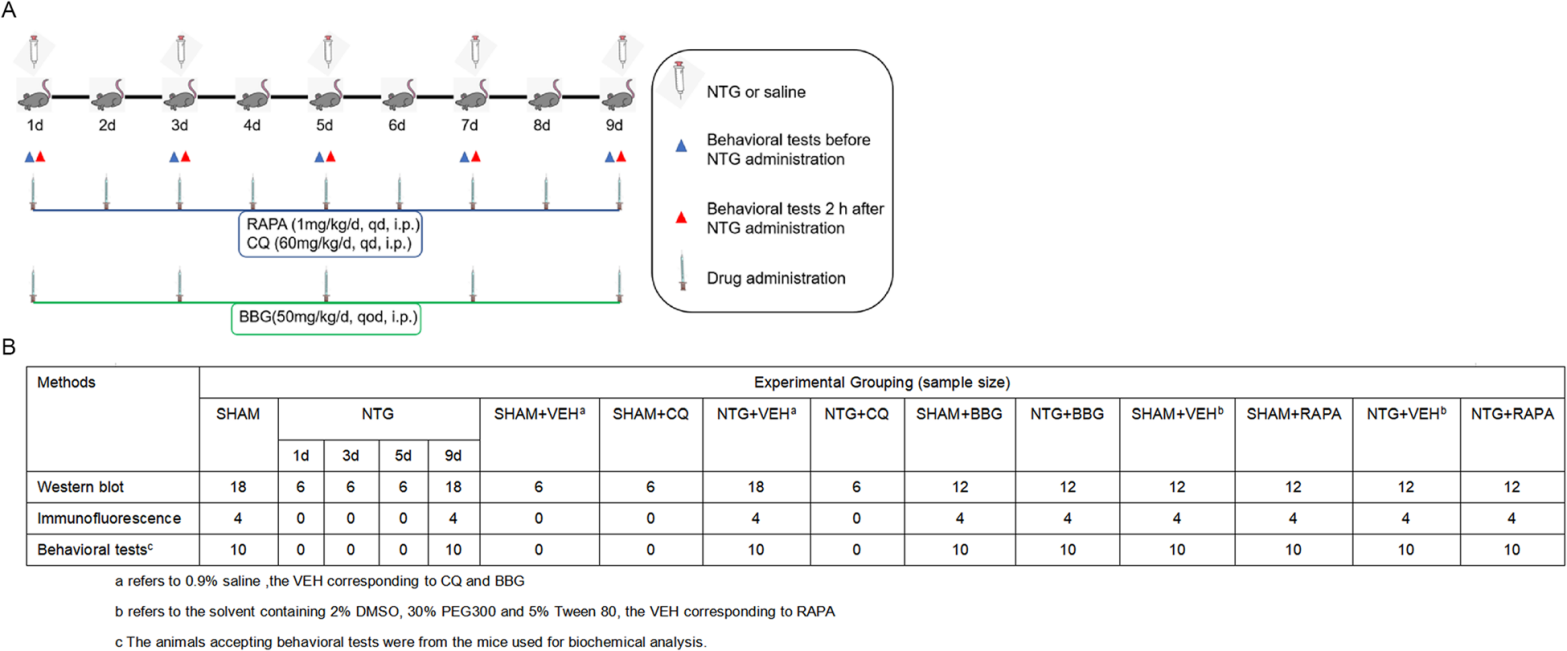
Experimental protocol for drug administration. **a** Schematic timeline diagram of drug treatment and behavioral assessment in mice. NTG: nitroglycerin. RAPA: rapamycin. CQ: chloroquine. BBG: Brilliant Blue G. **b** A table showing the experimental groups and sample sizes of mice per group. NTG: nitroglycerin. RAPA: rapamycin. CQ: chloroquine. BBG: Brilliant Blue G. VEH: vehicle

Male C57BL/6 mice weighing 20–30 g were used in this study. All animals were obtained from the Experiment Animal Center of Chongqing Medical University (Chongqing, China). Mice were kept in the standard experimental environment at 22 ± 2 °C and 50 ± 5% relative humidity with an alternating 12/12-h light/dark cycle. Food and water were provided ad libitum. Before the experiment, all the animals were acclimatized to the environment for at least 1 week and then be randomly assigned to different experimental groups on the basis of a random number sequence generated by Excel Software. The detailed procedure is illustrated as follows: (1) mice fulfilling the experimental criteria were selected and numbered. (2) Animal numbers were input into an Excel table, and a random sequence was generated using the “rand” function in Excel software; (3) the animal numbers were rearranged according to the ascending order of random sequence; (4) based on the new order, experimental animals were assigned to receive different treatments, with six mice in each group. Since the model induces pain in animals, the number of mice used was the minimum necessary to achieve sufficient statistical power. Figure 1b shows the detailed information about experimental group and sample size of mice for each experiment. The experimental protocol was approved by the Animal Care and Use Committee at Chongqing Medical University in China. All animal procedures were conducted in accordance with the National Institutes of Health Guide for the Care and Use of Laboratory Animals.

### Establishment of the chronic migraine model

The CM model was established as the previously described [29]. Nitroglycerin (NTG) (Beijing Regent, China) was prepared from a stock solution of 5.0 mg/ml NTG dissolved in 30% alcohol, 30% propylene glycol, and water. Before injection, NTG was freshly diluted to 1 mg/ml with 0.9% saline. Solution containing 0.9% saline, 6% propylene glycol, and 6% alcohol was used as a vehicle control. The animals intraperitoneally (i.p.) administered of 10 mg/kg of NTG or an equal volume of vehicle every second day for 9 days (five times in total) (Fig. 1a). All experimental animals were subjected to the behavioral tests described in detail below before and 2 h after NTG injection (Fig. 1a).

### Drug administration

To explore the level of autophagic flux in CM, 60 mg/kg chloroquine (CQ, MedChemExpress/MCE, American) was intraperitoneally injected once a day for 9 consecutive days, before NTG treatment and after baseline threshold measurement (Fig. 1a). To investigate the role of autophagy in CM, the autophagy inducer, rapamycin (RAPA, Selleck, TX, USA), was delivered in the same manner at a concentration of 1 mg/kg (Fig. 1a). The P2X7R selective antagonist, Brilliant Blue G (BBG, Sigma-Aldrich, Hungary), was used to figure out the function of P2X7R in CM. BBG was intraperitoneally administered at a dose of 50 mg/kg every other day for 9 days, in an identical manner to NTG treatment (Fig. 1a). CQ and BBG were diluted in 0.9% saline. The RAPA was dissolved in 2% DMSO, 30% PEG 300, and 5% Tween 80. An equivalent volume of solvent corresponding to each drug was used as a vehicle control. The drug dosage and methods of delivery were determined by literature data and preliminary experiments [16, 30, 31]. All drug solutions were freshly prepared on the day of use.

### Behavioral assessment

In the clinic, central sensitization is manifested as cutaneous allodynia and expansion of the pain area, including the craniofacial and non-craniofacial region [32]. Therefore, in the animal model, we measured both the periorbital and hind paw withdrawal threshold to mechanical stimulation, as performed in the previous references. The plantar thermal sensitivity was also evaluated, since chronic NTG delivery induces thermal hyperalgesia [33]. All the behavioral assessments were performed between 9:00 am to 15:00 pm in a quiet environment. Mice underwent a 3-day training period before the experiment. All the behavioral tests including mechanical withdrawal threshold and thermal withdrawal latency were performed in the same sets of animals. At the end of the experiment, six mice with successful modeling and significant behavioral tests were selected for further biochemical and morphological analyses. The behavioral testing was performed by the same investigators, who were blinded to the treatment groups.

### Measurement of the mechanical withdrawal threshold

The mechanical withdrawal threshold of the hind paw and periorbit was assessed every other day before and 2 h after NTG injection (Fig. 1a). We used Von Frey filaments with the up-down method to determine the withdrawal threshold to mechanical stimulation as previously described [7]. Briefly, a series of Von Frey filaments (range from 0.01 g to 2 g) were applied to the hind paw or periorbit, with an initial stimulation strength of 0.4 g. If there was no response to the stimulation, the filament strength was increased; otherwise, the filament strength was decreased until there was a positive reaction. Each filament was held for 5 s at the testing site with an interval of 1 min. The threshold was recorded as the lowest force evoking a positive response and averaged from three repetitive measurements.

For the hind paw test, the mice were separately placed in a suspended acrylic chamber covered with a wire mesh floor. The animals were acclimated to the new environment for at least 30 min. The Von Frey filaments were applied perpendicularly to the central area of the hind paw surface. Brisk withdrawal, shaking, lifting, or licking of the testing paw were considered positive responses. For the periorbital assessment, the mice were placed individually in a 4-oz. paper cup, allowing only the head to poke out. The head and fore paw could move freely, but body could not turn in the cup. The periorbital region included the area from caudal of eyes to approximately the midline. Vocalization, quick retraction of head from the stimulation, or scratching of face with the ipsilateral fore paw were considered positive responses.

### Measurement of thermal withdrawal latency

The thermal withdrawal latency of the hind paw was assessed every other day before and 2 h after NTG injection (Fig. 1a). We used a plantar test apparatus (Techman PL-200, Chengdu, China) with an intensity adjustable radiant heat to assess the thermal sensitivity. Briefly, mice were placed separately in a transparent chamber with a temperature controlled glass floor. After 30 min of acclimatization, the radiant thermal stimulus was delivered to the central part of the hind paw through the glass. The stimulus was shut off once the hind paw moved and the thermal withdrawal latencies were recorded automatically. The radiant heat intensity was calibrated to produce a basal withdrawal latency of 8–10 s in the control group. The cut-off time was 20 s to prevent tissue damage [33]. The radiant heat was delivered three times to each hind paw with an interval of 5 min. The thermal withdrawal latencies were defined as the average of three recordings.

### Western blot analysis

Mice were sacrificed under anesthesia with 10% chloral hydrate. The TNC was collected immediately and stored at − 80 °C. Tissues were homogenized in cold RIPA lysis buffer (Beyotime, Shanghai, China) containing a protease inhibitor, phenylmethylsulphonyl fluoride (PMSF, Beyotime, Shanghai, China) at 4 °C for 1 h. The lysate was centrifuged at 12000×*g* for 15 min in 4 °C, and then the protein concentration of the supernatant was determined with a BCA protein assay kit (Beyotime, Shanghai, China). The protein was denatured by heating at 100 °C for 5 min and stored at − 80 °C. Equal amounts of tissue protein (40 ug) were separated on 10% or 12% sodium dodecyl sulfate-polyacrylamide (SDS-PAGE) gels (Beyotime, Shanghai, China), and electrotransferred to polyvinylidene difluoride (PVDF) membranes (Millipore, USA). The membranes were blocked in Tris-buffered saline with Tween-20 (TBST buffer) containing 5% non-fat milk for 2 h at room temperature and incubated overnight at 4 °C with the following primary antibodies: rabbit anti-P2X7R, rabbit anti-CGRP, mouse anti-c-fos, rabbit anti-LC3, rabbit anti-SQSTM1/p62, rabbit anti-beclin1, rat anti-NLRP3, rabbit anti-IL-1β, rabbit anti-IL-18, and mouse anti-β-actin. The detailed information for all the antibodies used in this study is provided in Table 1. All the primary antibodies were diluted with special diluent (Beyotime, Shanghai, China). The next day, the membranes were washed three times with TBST for 10 min each and incubated with corresponding horseradish peroxidase conjugated secondary antibodies, including goat-anti-rabbit, goat-anti-mouse and goat-anti-rat, for 1 h at room temperature. The immunoreacted bands were revealed with an ECL detection kit (Advansta Inc., USA), visualized and analyzed with an imaging system (Fusion, Germany). β-actin was used to normalize the relative expression of the target proteins in different groups. Each western blot was repeated at least six times, and the consistent results were obtained.

**Table 1:** Antibody used in western blotting analysis, immunofluorescence staining assays

Antibody	Manufacturer	Catalog number	Host	Dilution
**For western blot analysis**				
P2X7	Sigma-Aldrich, USA	P8232	Rabbit	1:3000
LC3	Bimake, USA	A5202	Rabbit	1:500
Beclin1	Cell signaling technology, USA	3495	Rabbit	1:1000
P62	Cell signaling technology, USA	5114T	Rabbit	1:1000
NLRP3	Abcam, UK	ab205680	Rat	1:1000
IL-1β	Bioss, China	bs-6319R	Rabbit	1:1000
IL-18	Abcam, UK	ab207323	Rabbit	1:1000
CGRP	Abcam, UK	Ab139264	Rabbit	1:3000
c-fos	Santa Cruz, USA	sc-447	Mouse	1:1500
β-actin	ZSGB-BIO, China	TA-09	Mouse	1:3000
HRP^a^ conjugated anti-rabbit	ZSGB-BIO, China	ZB-2301	Goat	1:9000
HRP conjugated anti-mouse	ZSGB-BIO, China	ZB-2305	Goat	1:5000
HRP conjugated anti-rat	ZSGB-BIO, China	ZB-2307	Goat	1:5000
**For immunofluorescence staining**				
P2X7	Sigma-Aldrich, USA	P8232	Rabbit	1:500
CGRP	Santa Cruz, USA	sc-57053	Mouse	1:100
c-fos	Novus Biologicals, USA	NBP2-50057SS	Rabbit	1:5000
Iba1	Wako, Japan	019-19741	Rabbit	1:800
Iba1	Novus Biologicals, USA	NB100-1028SS	Goat	1:800
GFAP	Santa Cruz, USA	sc-33673	Mouse	1:200
NeuN	Abcam, UK	ab104224	Mouse	1:500
Cy3 Goat anti-rabbit IgG	Beyotime, China	A0516	Goat	1:500
Alexa Fluor 488 Goat anti-mouse IgG	Beyotime, China	A0428	Goat	1:500
Alexa Fluor 555 Donkey anti-rabbit IgG	Beyotime, China	A0453	Donkey	1:500
IFkine Green, Donkey anti-goat IgG	Abbkine, USA	A-24231-1	Donkey	1:400

^a^*HRP* horseradish peroxidase

### Immunofluorescence staining

To detect c-fos, tissues were collected 2 h after the last NTG or vehicle injection, while for other targets, tissues were collected within 12 h. Mice were anesthetized with 10% chloral hydrate and perfused transcardially with 60 ml of cold phosphate-buffered saline (PBS, pH 7.4) followed by 60 ml of 4% cold paraformaldehyde (PFA) in 0.1% PBS (pH 7.4). The brainstem and cervical spinal cord (C1–C2) were harvested and postfixed in 4% PFA for 24 h at 4 °C. The medullary segment containing the TNC between + 1 and – 3 mm from the obex was removed and dehydrated sequentially in 20% and 30% sucrose until the tissue sank. Tissues were embedded, flash frozen with 2-methylbutane (Aladdin, Shanghai, China) and sliced coronally into 10 um sections with a cryostat (Leica, Japan). After antigen retrieval with sodium citrate (Beyotime, Shanghai, China), the sections were blocked and permeabilized simultaneously with 0.3% Triton X-100 (Beyotime, Shanghai, China) in 5% donkey or goat serum (Boster, Wuhan, China) for 30 minutes at 37 °C. Then, the sections were incubated overnight at 4 °C with the following primary antibodies: rabbit anti-P2X7R, mouse anti-CGRP, rabbit anti-c-fos, rabbit anti-Iba1, goat anti-Iba1, mouse anti-NeuN, and mouse anti-GFAP. The detailed information for all the antibodies is provided in Table 1. All the primary antibodies were diluted with blocking solution. After rinsing three times for 15 min in PBS, the sections were reacted with corresponding secondary antibodies (conjugated to Alexa Fluor 488, 555 or cy3) for 90 min at 37 °C. Nuclei were stained with 4′,6-diamidino-2-phenylinodole (DAPI) (Beyotime, Shanghai, China) for 10 min at 37 °C. Images were captured with a confocal microscope (LSM800, ZEISS, Germany). Negative control sections were treated with PBS instead of primary antibody and showed no positive signals.

### Immunofluorescence imaging data analysis

The TNC area was determined based on the morphology under a light microscope according to the Mouse Brain Atlas [34]. The mean optical density of CGRP was analyzed using ImageJ software (version 1.8.0_112) with a × 10 objective. To quantify the number of immunopositive cells of Iba1 and c-fos, the squared images (field of view, FOV, 320 × 320 um^2^) in the superficial layer of the TNC (Fig. 4f) were taken at ×200 magnification. Four to six FOV per section were investigated. ImageJ software (version 1.8.0_112) was used to count the immunoreactive cells. To analyze microglial morphology, Neuron J, an ImageJ plug-in, was used to determine the total and mean length of microglial processes. Each cohort consisted of 4 mice, and 6–8 sections from each mouse were analyzed. The image collection and analysis were performed by an experimenter who was blinded to the treatment groups.

### Statistical analysis

GraphPad Prism version 7.0 (GraphPad Software Inc., San Diego, CA, USA) was used for the statistical analysis and graph generation. All the data are presented as the mean ± standard error of the mean (SEM). The Kolmogorov-Smirnov test and Bartlett’s tests were used to analyze the normality and homogeneity of the data, respectively. Differences between two groups were determined by an independent-sample *t* test. Comparison among three or more groups were investigated by one-way analysis of variance (ANOVA) followed by Tukey’s or Dunnett’s multiple comparison tests to detect pair-wise between-group differences. Behavioral data were analyzed by two-way ANOVA with the Bonferroni post hoc test. Two-tailed tests were applied in all statistical analyses. *P* < 0.05 was considered to be statistically significant.

## Results

### Autophagic flux impairment in the TNC after chronic NTG administration

LC3-II is expressed in the autophagosome membrane and is widely used as a reliable autophagy marker since the protein level of LC3-II strictly reflects the number of autophagosomes [35]. The accumulation of LC3-II is usually caused by increased autophagosome formation due to enhanced autophagic flux or dysfunctional degradation of the autophagosome due to impaired autophagic flux [22, 28, 35]. The p62 protein is a well-known autophagy substrate that helps to determine the reason for the change in LC3-II, thus providing the additional evidence of autophagic flux [35]. The activated autophagy usually causes a decrease in p62, while the inhibited autophagy leads to a complete opposite effect [35, 36]. In addition, beclin1 is also a key protein reflecting the autophagy activity [35]. Therefore, we detected the expression of autophagy-related proteins, including LC3-II, beclin1, and p62, to evaluate the level of autophagic flux in the CM model.

**Fig. 2:**
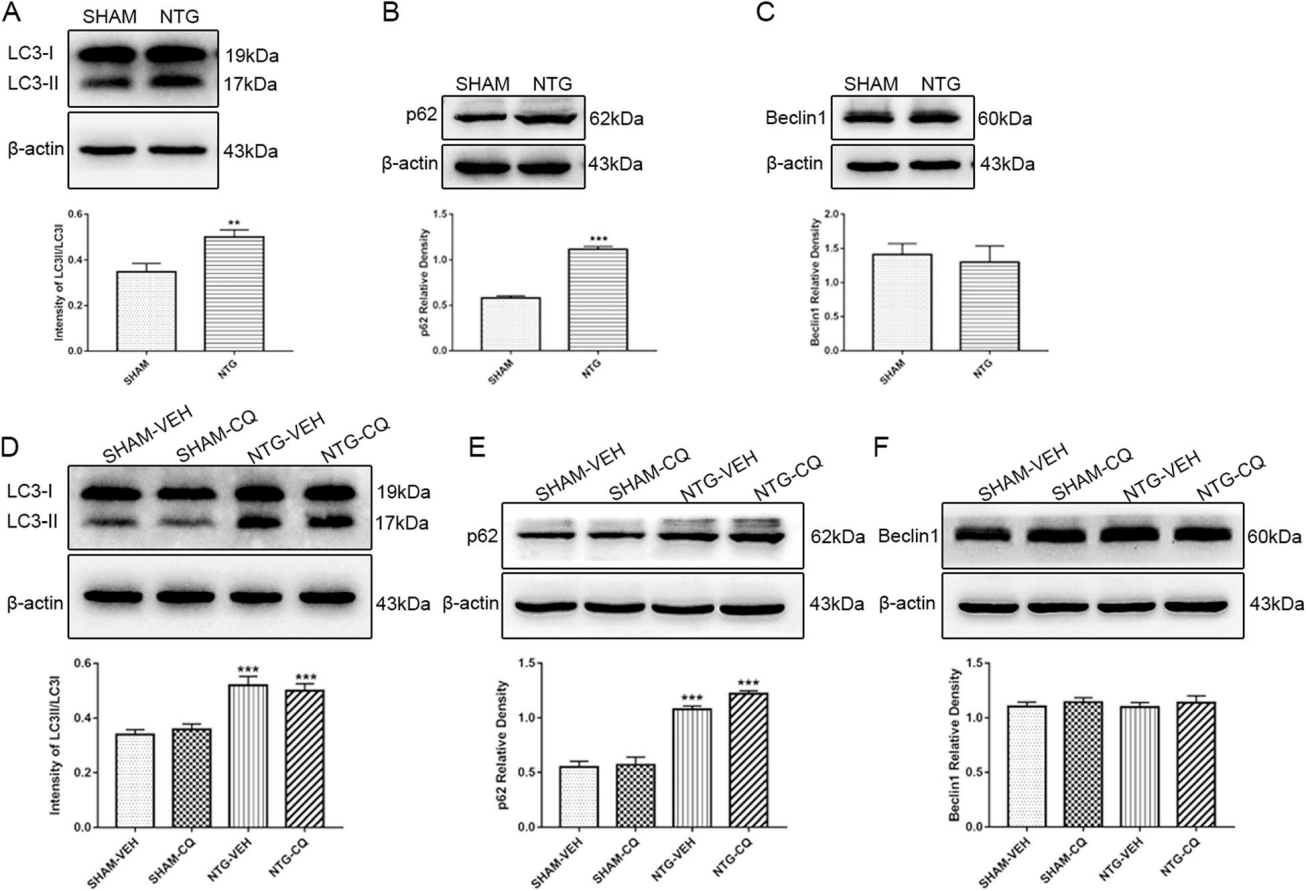
Autophagy impairment in the TNC after chronic NTG administration. **a**–**c** Representative western blot of autophagy-related proteins following NTG injection. Western blot assay revealed increased ration of LC3II/LC3I (**a**), in combination with the up-regulation of p62 (**b**), without any change in beclin1 (**c**) under the chronic stimulation with NTG. *n* = 6 per group; data are presented as mean ± SEM; independent-sample *t* test; **p < 0.01, ***p < 0.001 compared with the SHAM group; to further determine the autophagic flux, mice were treated with CQ, an inhibitor of lysosome function, every day for 9 days prior to NTG/VEH administration. **d**–**f** The representative immunoblot showing the change of autophagy-related proteins after CQ treatment. Quantitative analysis of band intensities showed that, after CQ treatment, LC3II did not further increase (**d**), the expression of p62 tended to increase, but the difference was not statistically significant (**e**), and the level of beclin1 did not change (**f**), which confirmed the inhibition of autophagic flux in CM model. *n* = 6 per group; values are expressed as mean ± SEM; one-way ANOVA and Tukey’s multiple comparison test; ***p < 0.001 compared with the SHAM-VEH group

As shown in Fig. 2, recurrent NTG treatment significantly increased the ration of LC3-II/LC3-I, augmented the expression of p62, and did not alter the level of beclin1, which indicated an impaired autophagic flux (Fig. 2a–c). To further confirm the change in autophagic flux, we intraperitoneally administered CQ to inhibit lysosome function by increasing the PH of the lysosomal cavity. The data showed that, compared with mice receiving NTG only, the combination treatment of CQ and NTG did not further increase the ratio of LC3-II/LC3-I (Fig. 2d). The expression of p62 had an increasing trend, but the difference was not statistically significant (Fig. 2e). The level of beclin1 was similar in mice treated with CQ and those without CQ (Fig. 2f). These combined data revealed an impaired autophagic flux in the TNC of the CM model caused by a dysfunctional degradation pathway of autophagy, thus leading to the upregulation of autophagy-related structural proteins.

### Autophagy induction prevented basal rather than acute mechanical and thermal hyperalgesia

**Fig. 3:**
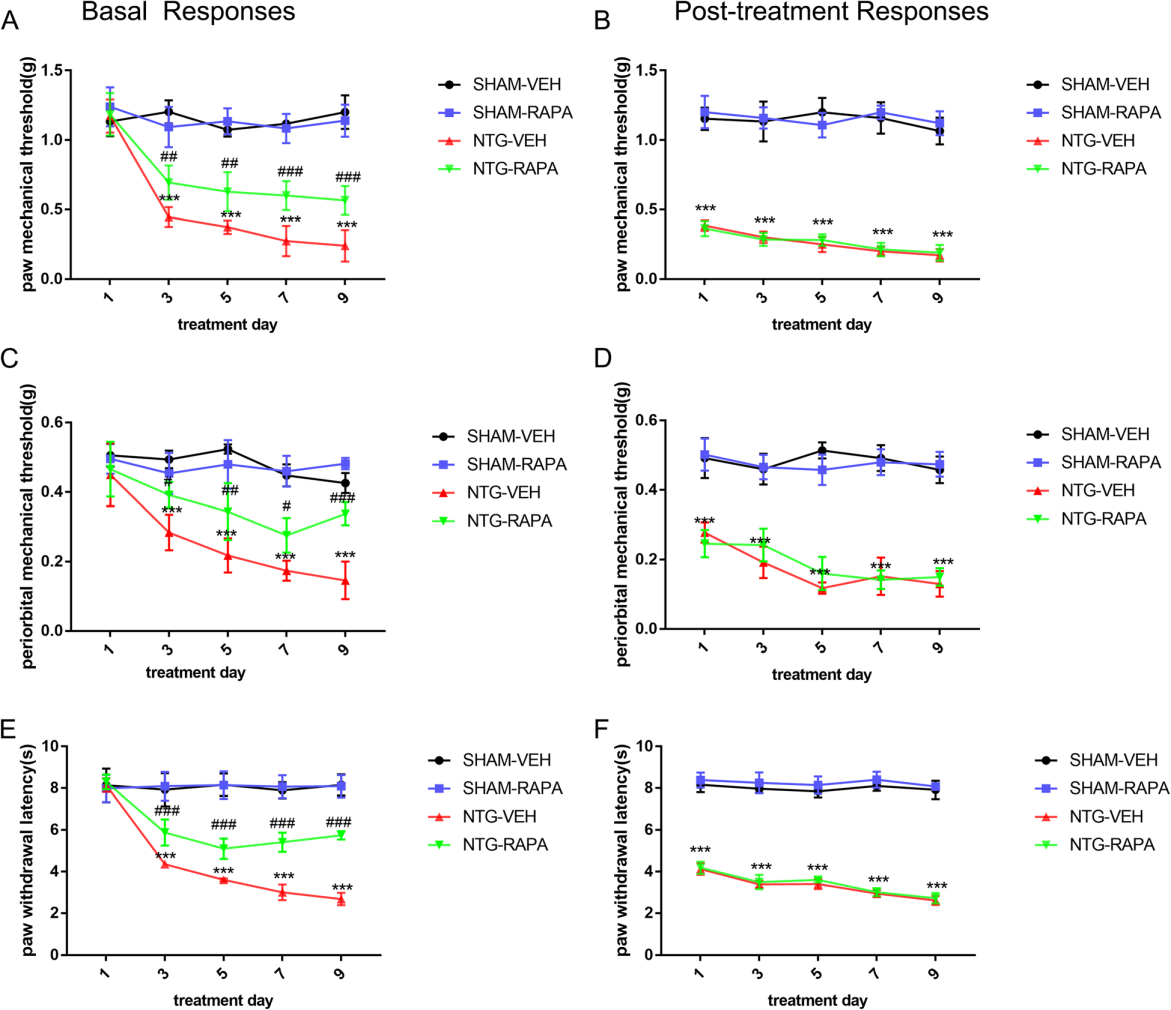
Autophagy activation attenuated NTG-induced basal, not acute, mechanical, and thermal hyperalgesia. **a**, **c**, **e** Repeated administration of RAPA, the autophagy inducer, improved basal mechanical hyperalgesia of the hind paw (**a**), periorbit (**c**), and thermal hyperalgesia of the hind paw (**e**). **b**, **d**, **f** RAPA did not prevent the post-treatment responses comprising hind paw mechanical hypersensitivity (**b**), periorbital mechanical hypersensitivity (**d**), and thermal hyperalgesia (**f**). *n* = 10 per group; data are presented as the mean ± SEM; two-way ANOVA and Bonferroni post hoc test were performed; ***p < 0.001 compared with the SHAM-VEH group; ^#^*p* < 0.05, ^##^*p* < 0.01, ^###^*p* < 0.001 compared with the NTG-VEH group

In line with previous studies, chronic NTG injection induced a gradual decrease in the basal pain threshold over time, and each single NTG treatment produced an acute reduction of the pain threshold after 2 h. To determine the role of autophagy in CM, we used RAPA, an autophagy inducer, to activate autophagy and evaluated the effect of RAPA on NTG-induced hyperalgesia. The RAPA (NTG-RAPA) markedly attenuated the basal mechanical and thermal hyperalgesia, while it had no effect on the post-treatment response (Fig. 3). Only RAPA treatment (SHAM-RAPA) did not alter the pain threshold. These data suggested that autophagy in the TNC exerted a beneficial effect against CM development. The autophagy activation contributed greatly to the reduction of CM-liked pain behavior.

### Activation of autophagy reduced CGRP and c-fos expression in the TNC after recurrent NTG administration

**Fig. 4:**
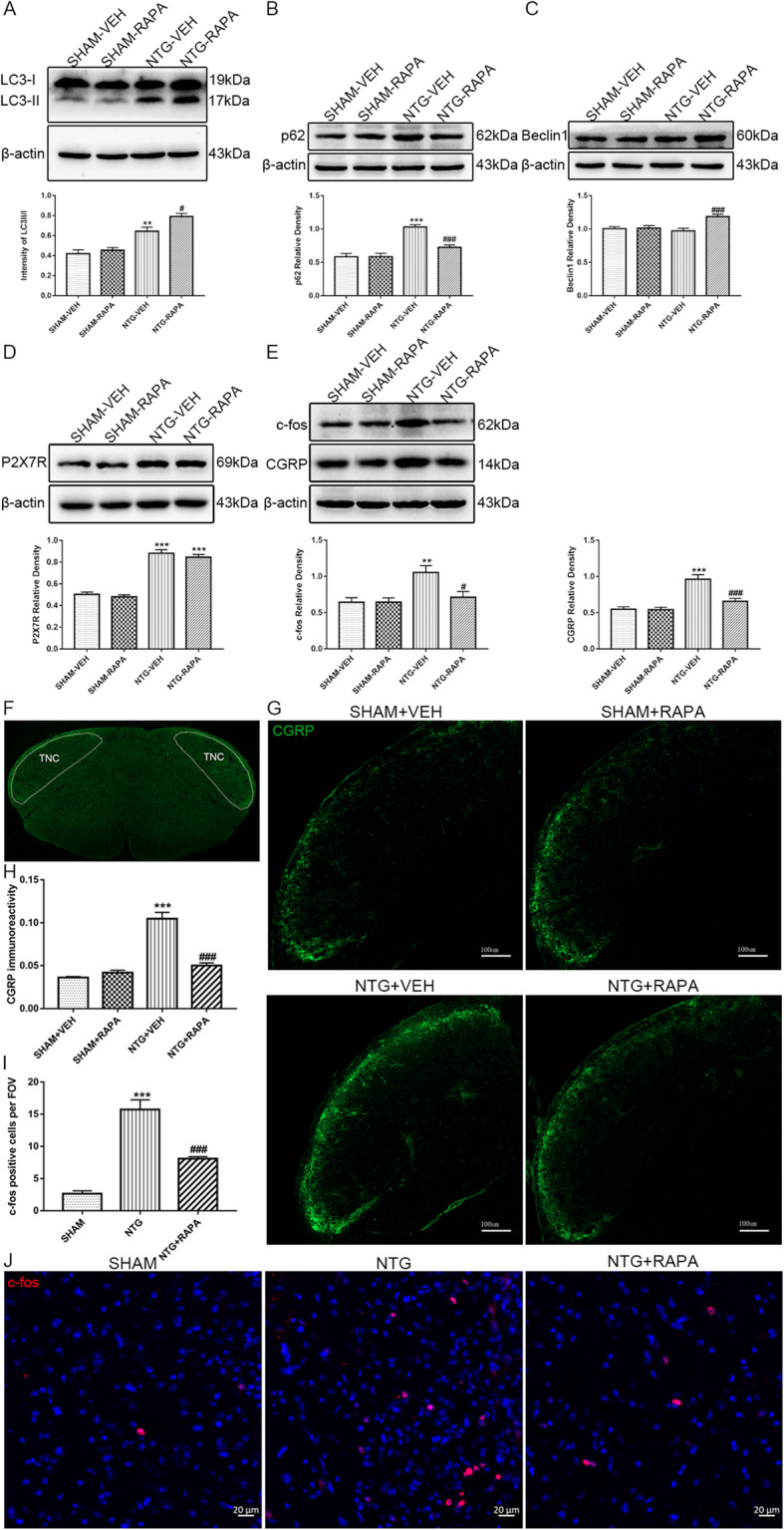
Activation of autophagy reduced NTG-induced CGRP and c-fos expression in the TNC. **a–c** Representative western blots showing the change of autophagy-related proteins after RAPA treatment. Quantification of band intensities revealed increased ration of LC3II/LC3I (**a**), in combination with decreased p62 (**b**), as well as upregulated beclin1 (**c**) after chronic RAPA and NTG administration compared with NTG administration only. **d** Immunoblots of P2X7R showed that autophagy activation did not affect the expression of P2X7R. **e** Representative immunoblots of CGRP and c-fos after RAPA intervention. The optical density analysis indicated RAPA treatment significantly decreased the expression of CGRP and c-fos compared with NTG administration only. *n* = 6 per group; data are presented as mean ± SEM; one-way ANOVA and Tukey’s multiple comparison test; **p < 0.01 and ***p < 0.001 compared with the SHAM-VEH group; ^#^*p* < 0.05 and ^###^*p* < 0.001 compared with the NTG-VEH group. **f** The white dotted line frame indicated the representative image of the TNC area used for the analyses. **g**, **j** Immunofluorescence images of CGRP (**g**) and c-fos (**j**) in the TNC area. Scale bars: 100 μm for CGRP, 20 μm for c-fos. **h**, **i** The CGRP immunoreactivity (**h**) and c-fos-positive cells (**i**) were reduced by RAPA treatment. *n* = 4 per group; 6 sections from each mouse were analyzed; values are expressed as mean ± SEM; one-way ANOVA and Tukey’s multiple comparison test; ***p < 0.001 compared with the SHAM-VEH group for CGRP or the SHAM group for c-fos ; ^###^*p* < 0.001 compared with the NTG-VEH group for CGRP or the NTG group for c-fos

To investigate the effect of autophagy on central sensitization, mice were injected intraperitoneally with RAPA (1 mg/kg/day) to activate autophagy, once a day for 9 days. We found the up-regulated ration of LC3-II/LC3I in combination with decreased expression of p62 and an elevated protein level of beclin1 in mice with RAPA treatment, compared with mice administered NTG only (Fig. 4a–c). The expression changes in autophagy-related proteins are consistent with characteristics of activated autophagy. No difference was found in P2X7R expression between modeling mice treated with and without RAPA (Fig. 4d), which indicated that autophagy acted downstream of P2X7R in the TNC. CGRP is a pivotal endogenous neuropeptide involved in the initiation and development of CM. A large number of studies have shown that CGRP plays an important role in central sensitization [37, 38]. Western blot showed that the protein level of CGRP in mice pretreated with RAPA was lower than that in mice treated with NTG (Fig. 4e). The mean optic density (OD) of CGRP immunoreactive fibers in the superficial layers of the TNC was also reduced by RAPA delivery (Fig. 4g, h). c-fos is recognized as a reliable marker of neuronal activation to nociceptive stimulation. The increased expression of c-fos in the trigeminal pain pathway suggests the activation of nociceptive neurons, which is closely related to central sensitization [39]. Consistent with our previous studies, the protein level of c-fos was upregulated following NTG injection (Fig. 4e). The number of c-fos-positive cells in the superficial layer of the TNC also increased (Fig. 4i, j). Pretreatment with RAPA attenuated the NTG-induced expression of c-fos both in western blot grayscale analysis and immunofluorescence quantitative analysis (Fig. 4e, i, j). These combined data suggested that the activated autophagy alleviated central sensitization of CM.

### Autophagy induction attenuated the NTG-evoked activation of microglia and NLRP3 inflammasome

**Fig. 5:**
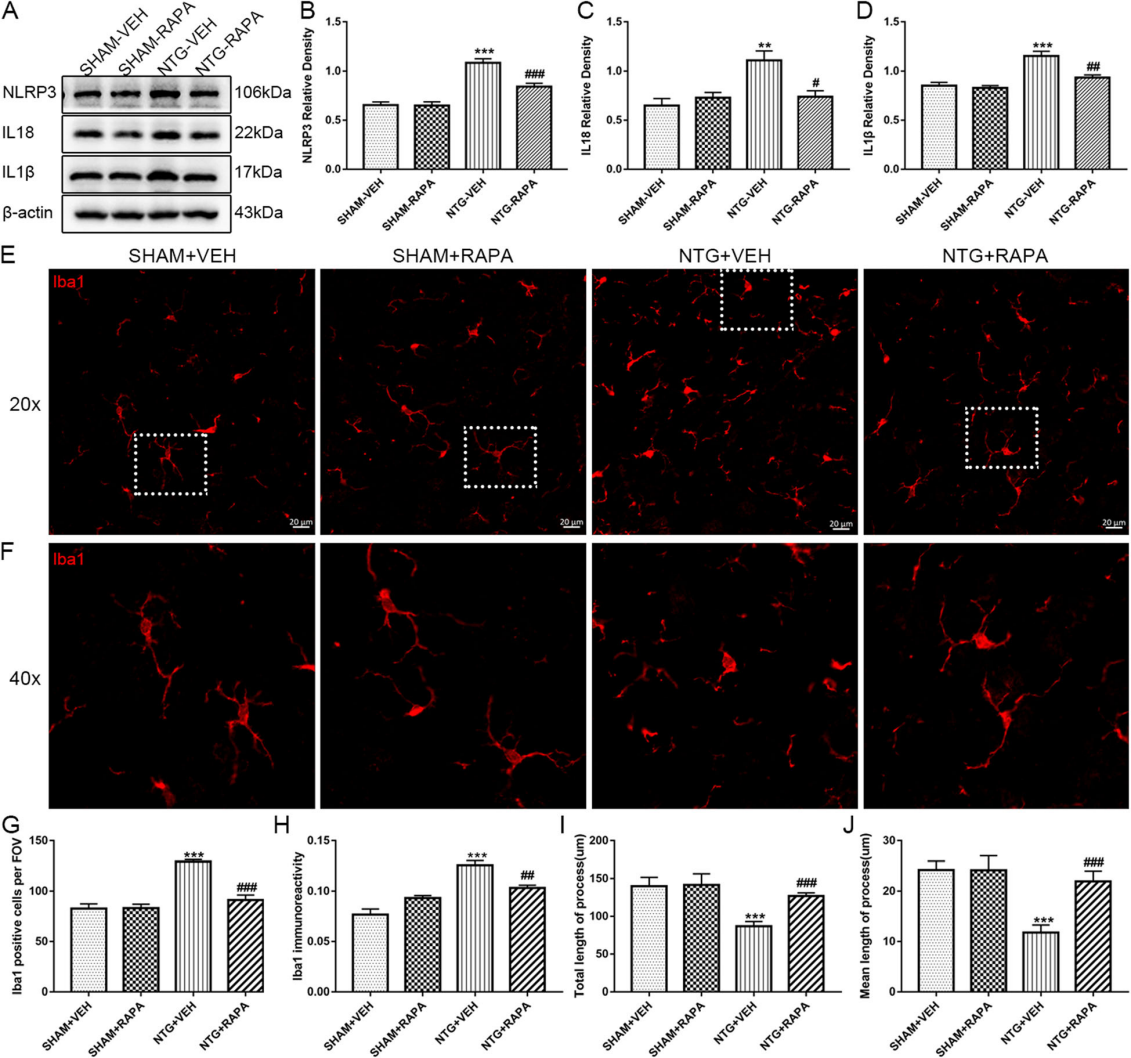
Autophagy induction inhibited the activation of microglia and the NLRP3 inflammasome. **a** Representative western blot of NLRP3, IL-1β, and IL-18 expression in the TNC after NTG, RAPA administration. β-actin was used as the control and band density normalization. **b–d** The optical density analysis of NLRP3 (**b**), IL-18 (**c**), IL-1β (**d**). The data showed that RAPA significantly reduced the expression of NLRP3 (**b**), IL-18 (**c**), IL-1β (**d**). *n* = 6 per group; data are presented as mean ± SEM; one-way ANOVA and Tukey’s multiple comparison test; **p < 0.01, ***p < 0.001 compared with the SHAM-VEH group; ^#^*p* < 0.05, ^##^*p* < 0.01, and ^###^*p* < 0.001 compared with the NTG-VEH group. **e** Immunostaining of Iba1 in the TNC of different groups at × 200 magnification. Scale bar: 20 μm. **f** Magnified images (× 400 magnification) of Iba1 in the areas of the white dotted line frame in **e. g**–**j** Quantitative analysis of the Iba1-positive cells (**g**), Iba1 immunoreactivity (**h**), total (**i**), and mean (**j**) length of processes per microglia. *n* = 4 per group; 6 sections from each mouse and 4–6 FOV from each section were analyzed; values are expressed as mean ± SEM; one-way ANOVA and Tukey’s multiple comparison test; ***p < 0.001 compared with the SHAM-VEH group; ^##^*p* < 0.01 and ^###^*p* < 0.001 compared with the NTG-VEH group

Previous studies have shown that autophagy regulates the activation of microglia and inflammatory response [40, 41]. The activation of NLRP3 inflammasome is inhibited by autophagy through the clearance of damaged mitochondria and degradation of the NLRP3 inflammasome [26]. Therefore, we used western blot analysis to detect the expression of NLRP3 and related inflammatory factors, IL-18 and IL-1β. Immunofluorescence staining was used to analyze changes in microglial quantity, immunoreactivity, and morphology. Consistent with our previous studies, NTG treatment significantly increased the expression of NLRP3, IL-18, and IL-1β in the TNC (Fig. 5a–d). Immunofluorescence staining of Iba1 showed that the number of Iba1-positive cells increased with enhanced immunoreactivity after NTG administration (Fig. 5e–h). Morphologically, the microglia presented with hypertrophied cell bodies and shorter and fewer processes. The quantitative analysis showed that the total and mean length of processes decreased (Fig. 5e, f, i ,j). Mice receiving RAPA treatment exhibited a significant decrease in the expression levels of NLRP3, IL-18, and IL-1β, compared to mice receiving NTG only (Fig. 5a–d). In addition, RAPA pretreatment inhibited microgliosis, reduced Iba1 immunoreactivity, and changed the microglia into a ramified shape, exhibiting increases in the total and mean length of processes (Fig. 5e–j). Taken together, these results revealed that enhanced autophagic flux suppressed the activation of microglia and the NLRP3 inflammasome.

### P2X7R was increasingly expressed in the TNC area after recurrent NTG administration

**Fig. 6:**
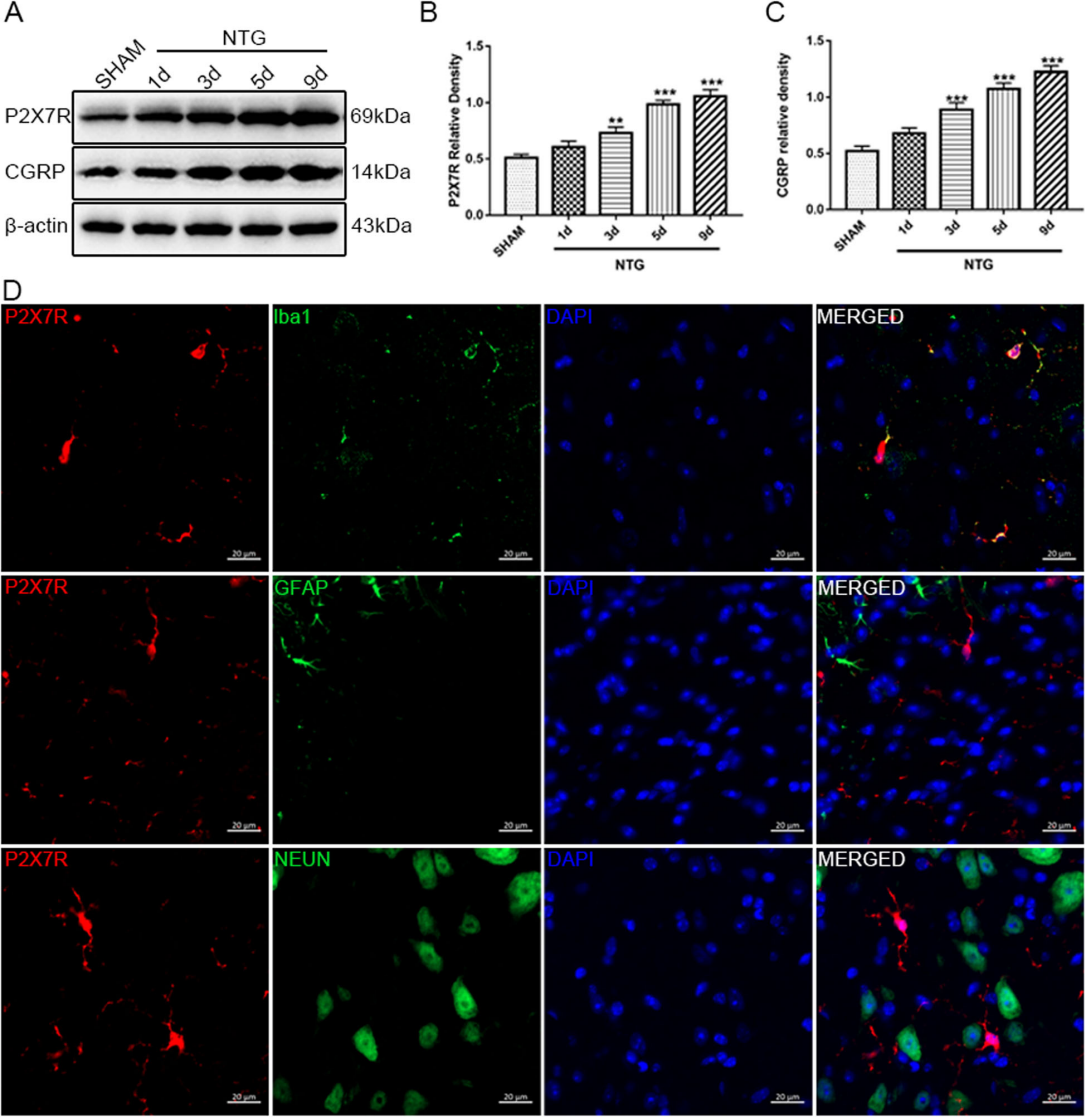
The microglial P2X7R increased in the TNC after chronic administration of NTG. **a** Representative western blot of P2X7R and CGRP on different time points in the TNC. **b**, **c** The band intensities of P2X7R and CGRP relative to those of β-actin indicated that the protein level of P2X7R (**b**) and CGRP (**c**) increased gradually with the development of CM model. *n* = 6 per group; data are presented as mean ± SEM; one-way ANOVA and Dunnett’s multiple comparison test; **p < 0.01 and ***p < 0.001 compared with the SHAM group. **d** Double immunofluorescence staining of P2X7R and Iba1, GFAP, and NEUN in the TNC area. Most P2X7R-positive cells are double labeled with Iba1. Scale bar: 20 μm

To investigate the effect of NTG treatment on P2X7R expression, we monitored the protein level of P2X7R in the TNC at different time points during modeling. First, as shown in Fig. 6a, c, the expression of CGRP increased gradually with the development of modeling, which was consistent with the hyperalgesia induced by NTG and suggested a successful CM model. The expression level of P2X7R continuously rose in a time-dependent manner following repeated intermittent NTG injection. Specifically, the P2X7R protein level significantly increased on the third day and peaked on the last day of NTG treatment (Fig. 6a, b). To determine the cell types expressing P2X7R in the TNC, we performed double immunofluorescence staining to label the P2X7R protein and cell type-specific markers, including Ibal-1 for microglia, NeuN for neurons, and GFAP for astrocytes. The images showed that P2X7R was mainly co-localized with Iba1, but not with NeuN or GFAP (Fig. 6d). The results indicated that P2X7R was mainly expressed in microglia. Collectively, these data revealed that recurrent NTG administration provoked a significant increase in microglial P2X7R in the TNC.

### Blockage of P2X7R partially prevented mechanical and thermal hyperalgesia

**Fig. 7:**
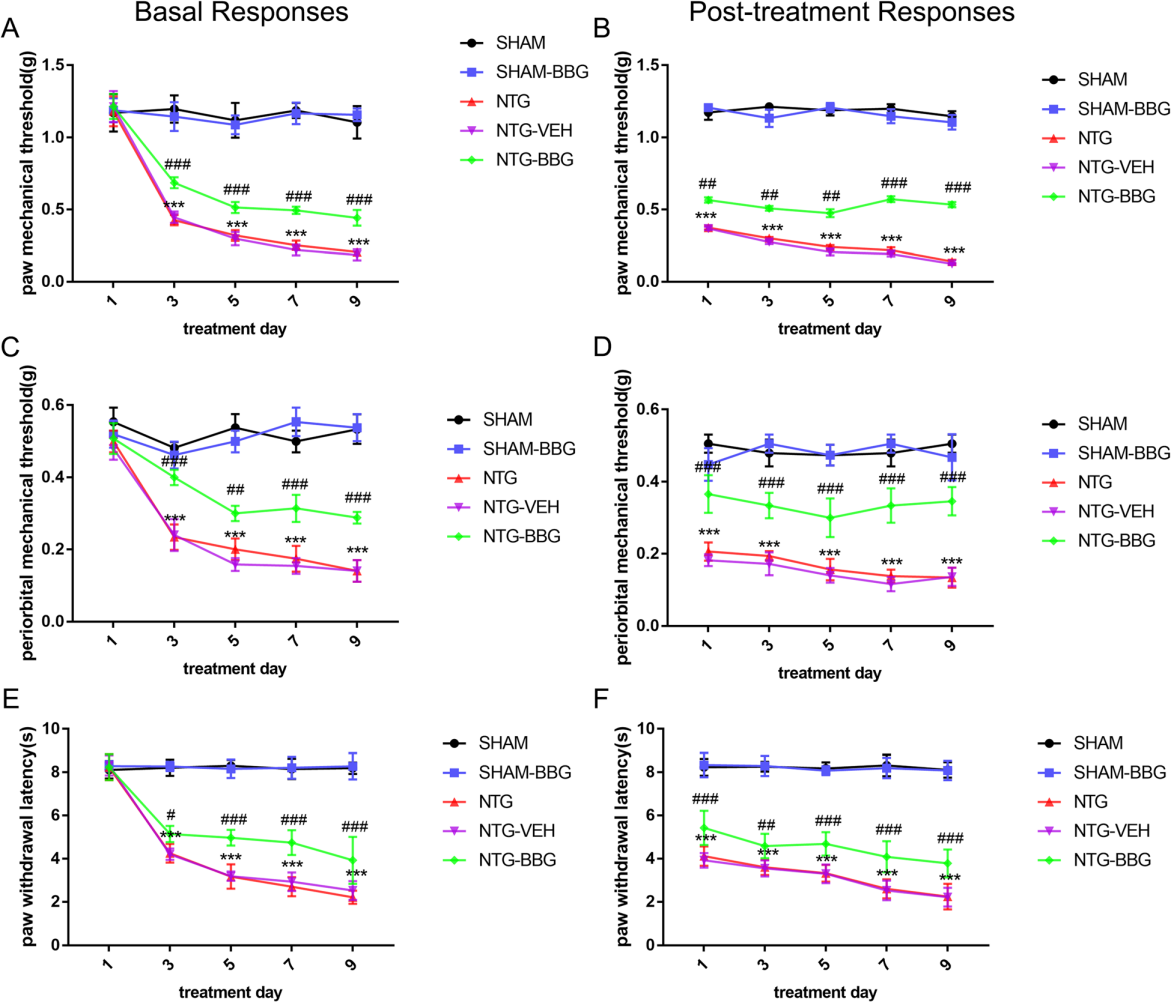
Inhibition of P2X7R ameliorated NTG-induced CM associated pain. Chronic treatment with BBG, the specific P2X7R antagonist, significantly relieved NTG-induced basal mechanical hyperalgesia of hind paw (**a**), and periorbit (**c**) and alleviated thermal hyperalgesia of hind paw (**e**). Post-treatment responses were assessed 2 h after NTG administration. BBG prevented NTG-induced acute pain hypersensitivity, including hind paw (**b**) and periorbital (**d**) mechanical hyperalgesia and thermal hyperalgesia (**f**). *n* = 10 per group; values are expressed as mean ± SEM; two-way ANOVA with a Bonferroni post hoc test were performed; ***p < 0.001 compared with the SHAM group; ^#^*p* < 0.05, ^##^*p* < 0.01, ^###^*p* < 0.001 compared with the NTG group

To determine whether P2X7R contributed to the development of CM, mice received an intraperitoneal injection of the specific P2X7R antagonist, BBG (50 mg/kg), prior to NTG injection every other day for 9 days. We found that BBG treatment notably increased the mechanical withdrawal threshold of the hind paw and periorbit, and prolonged the thermal withdrawal latency of the hind paw. In addition, BBG was effective in ameliorating both the basal and acute hyperalgesia (Fig. 7). Therefore, we concluded that P2X7R was involved in the initiation and development of CM. Blockage of P2X7R had a positive effect on alleviating CM-associated pain.

### P2X7R inhibition decreased CGRP and c-fos expression in the TNC after repetitive intermittent NTG treatment

**Fig. 8:**
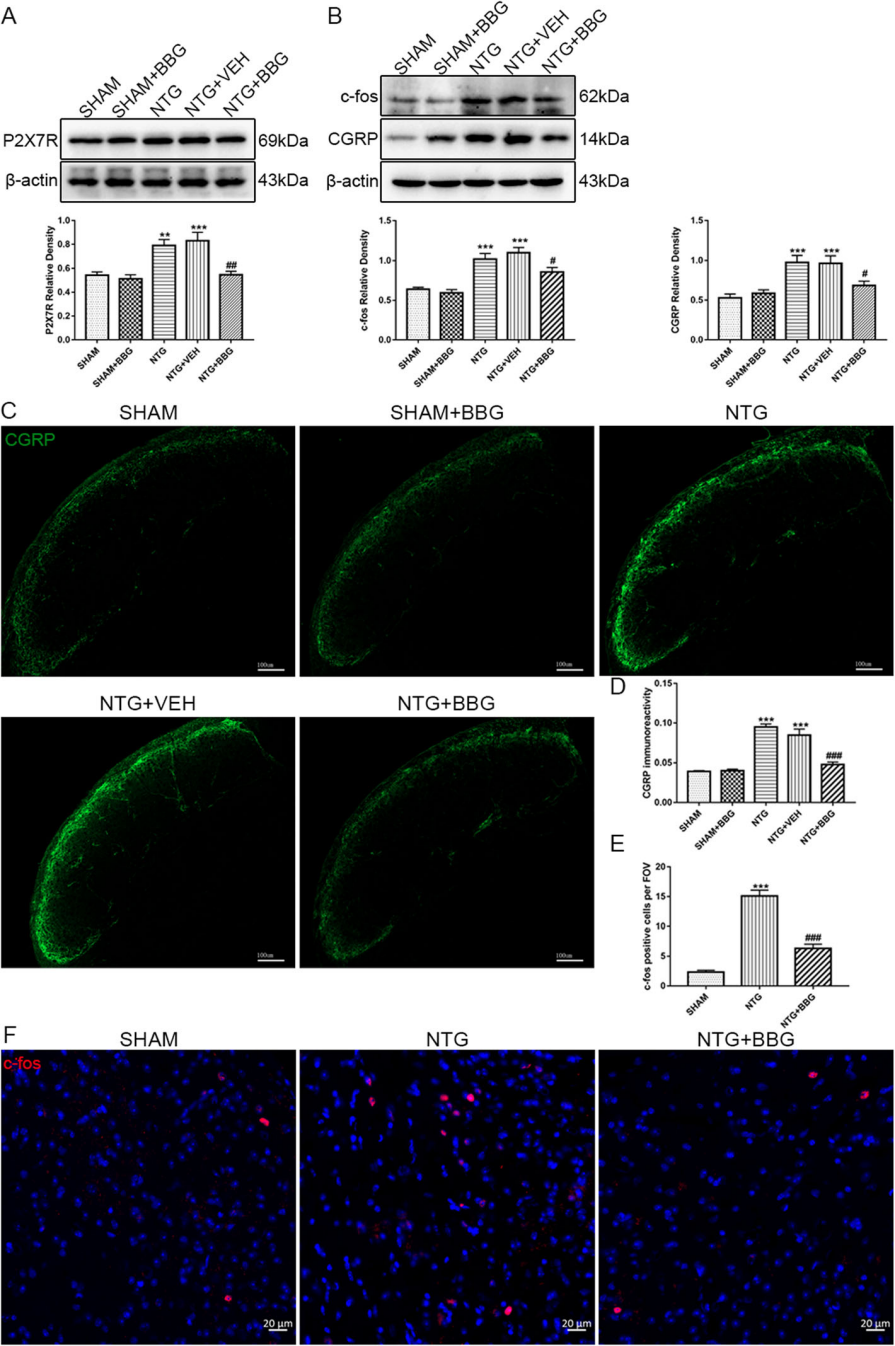
Inhibiting P2X7R reduced NTG-induced CGRP and c-fos expression in the TNC. **a** Western blots showing the P2X7R inhibitor, BBG, reduced the expression of P2X7R induced by NTG in the TNC. **b** Representative immunoblots of c-fos and CGRP in the TNC after BBG treatment. Quantification of band intensities revealed that BBG partially reversed the upregulation of c-fos and CGRP induced by NTG treatment. *n* = 6 per group; Data are presented as mean ± SEM; one-way ANOVA and Tukey’s multiple comparison test; **p < 0.01 and ***p < 0.001 compared with the SHAM group; ^##^*p* < 0.01 and ^###^*p* < 0.001 compared with the NTG group. **c**, **f** Immunofluorescence staining of CGRP (**c**) and c-fos (**f**) in the TNC area. Scale bars: 100 μm for CGRP, 20 μm for c-fos. **d**, **e** Quantification of CGRP immunoreactivity (**d**) and c-fos-positive cells (**e**) revealed consistent changes with the western blot analysis. *n* = 4 per group; 6 sections from each mouse were analyzed; values are expressed as mean ± SEM; one-way ANOVA and Tukey’s multiple comparison test; ***p < 0.001 compared with the SHAM group; ^###^*p* < 0.001 compared with the NTG group

To determine whether the upregulation of P2X7R contributed to central sensitization of CM, we delivered BBG and evaluated its effect on the expression of CGRP and c-fos. As shown by western blot analysis, we detected a remarkable decrease in CGRP and c-fos in the TNC homogenized tissue after P2X7R inhibition (Fig. 8b). Immunofluorescence staining showed that the immunoreactive intensity of CGRP fibers and the number of c-fos-positive cells were significantly reduced following BBG treatment, which was consistent with the results of western bolt analysis (Fig. 8c–f). These data supported the contribution of P2X7R to the pathophysiological mechanism of CM.

### Blockage of P2X7R activated autophagic flux in the TNC

**Fig. 9:**
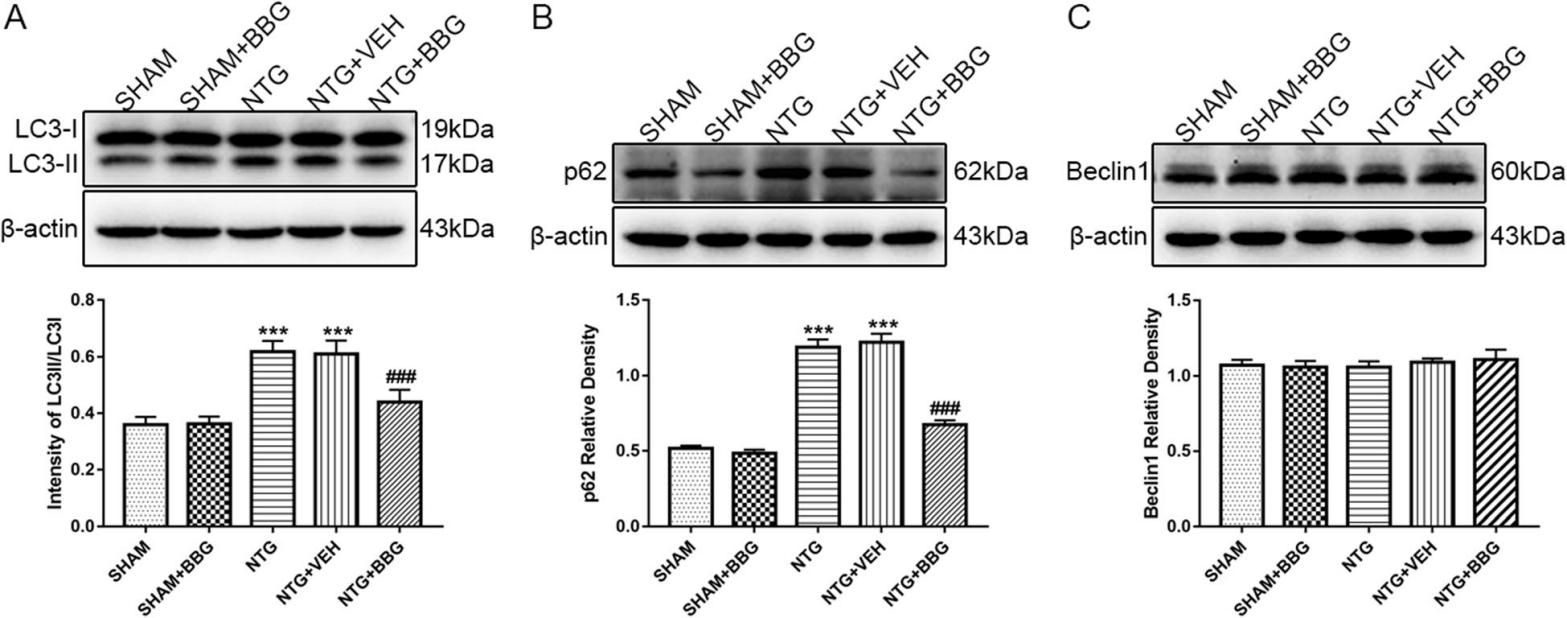
Blocking P2X7R promoted autophagic flux in the TNC after recurrent NTG injection. **a–c** Representative western blot of autophagy-related proteins after P2X7R inhibition. β-actin was used as the control and band density normalization. The optical density analysis showed that after BBG administration, the ratio of LC3II/LC3I (**a**) decreased, in combination with reduced expression of p62 (**b**), without any change in beclin1 (**c**). *n* = 6 per group; one-way ANOVA and Tukey’s multiple comparison test; data are presented as mean ± SEM; ***p < 0.001 compared with the SHAM group; ^##^*p* < 0.01 compared with the NTG group

Numerous studies have indicated the involvement role of P2X7R in autophagy regulation [20, 23]. Short-term P2X7R activation has been shown to stimulate autophagic flux, while persistent P2X7R stimulation leads to autophagic dysfunction [22]. Here, we assessed the effect of P2X7R on autophagic flux in the CM. The western blot analysis suggested that BBG treatment significantly decreased the LC3-II/LC3-I ratio and reduced the expression of p62. The protein level of beclin1 was similar between mice receiving both BBG and NTG and mice receiving NTG only (Fig. 9a–c). The results indicated that blocking P2X7R could enhance the level of autophagic flux in the TNC.

### P2X7R inhibition reduced the activation of microglia and the NLRP3 inflammasome

**Fig. 10:**
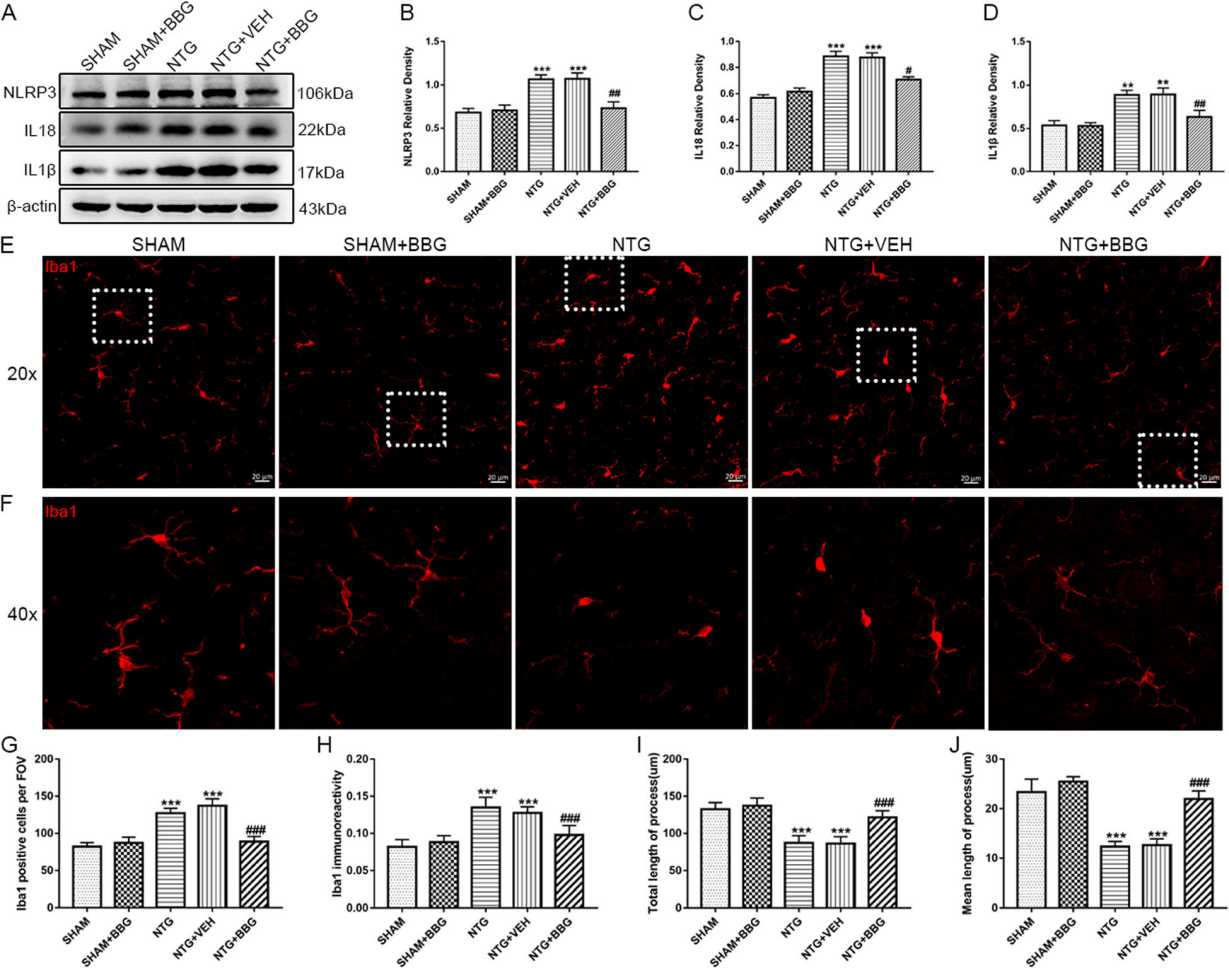
The P2X7R antagonist BBG inhibited the activation of microglia and the NLRP3 inflammasome. **a** Representative immunoblots of NLRP3, IL-18, and IL-1β expression in the TNC after treatment with NTG, BBG. **b**–**d** Quantification of NLRP3 (**b**), IL-18 (**c**), IL-1β (**d**) expression was relative to those of β-actin. Chronic administration of NTG significantly increased the protein level of the above three indexes. Pretreatment with BBG inhibited the NTG induced expression of NLRP3, IL-18, and IL-1β. *n* = 6 per group; one-way ANOVA and Tukey’s multiple comparison test; data are presented as mean ± SEM; **p < 0.01, ***p < 0.001 compared with the SHAM group; ^#^*p* < 0.05 and ^##^*p* < 0.01 compared with the NTG group. **e** Iba1 immunostaining in the TNC following NTG and BBG treatment. Scale bar: 20 μm. **f** Magnified images (× 400 magnification) of Iba1 in the areas of the white dotted line frame in **e**. **g**–**j** Quantification of the Iba1-positive cells (**g**), Iba1 immunoreactivity (**h**), total (**i**), and mean (**j**) length of processes per microglia. *n* = 4 per group; 6 sections from each mouse and 4–6 FOV from each section were analyzed; values are expressed as mean ± SEM; one-way ANOVA and Tukey’s multiple comparison test; ***p < 0.001 compared with the SHAM group; ^###^*p* < 0.001 compared with the NTG group

To elucidate the role of P2X7R in the regulation of microglia and NLRP3 inflammasome activation, we measured the expression of NLRP3 and related inflammatory factors using western blotting. Immunofluorescence was applied to detect the change in quantity and morphology of the microglia. We found that the levels of NLRP3, IL-1β, and IL-18 in mice receiving P2X7R antagonist were significantly lower than that in mice without P2X7R inhibition (Fig. 10a–d). After chronic pretreatment with BBG, both the Iba1 immunoreactivity and the number of positive cells were reduced compared with NTG group (Fig. 10e–h). Additionally, the total and mean length of the microglial processes were notably increased (Fig. 10e, f, i, j). Morphologically, BBG treatment changed the microglia into a ramified shape, which was similar to that in the SHAM group (Fig. 10e, f). Therefore, we concluded that P2X7R played a pivotal role in microglia and NLRP3 inflammasome activation.

## Discussion

Our current study provided several new findings as follows. First, we observed dysfunctional autophagic flux in the NTG-induced CM model and determined the beneficial effect of autophagy activation on pain relief for the first time. Subsequently, we confirmed that microglial P2X7R in the TNC was involved in the pathophysiological process of CM. In addition, we provided the first evidence from the in vivo experiment of CM that P2X7R activation promoted the activation of microglia and the NLRP3 inflammasome by negatively regulating the autophagic flux, thus contributing to the central sensitization. Our data demonstrated the regulation of autophagy as a novel mechanism by which P2X7R modulate the activation of microglia and the NLRP3 inflammasome.

In this study, we used repeated intermittent NTG injection to establish the CM animal model, which is now generally accepted as a reliable mice model of CM due to the similar features between mice with NTG injection and patients with CM [29]. First, NTG is a definite migraine trigger and is widely used in clinical trials [42, 43]. Studies have shown that a single treatment of NTG induces a significant decrease in the acute mechanical withdrawal threshold, while repeated injection of NTG evokes a progressive decrease in the basal mechanical withdrawal threshold that persists for 1 week after NTG cessation [43]. This phenomenon mimics the occurrence of cutaneous allodynia both in the ictal and interictal period of migraine in CM patients [44]. Additionally, the NTG-induced hyperalgesia can be relieved by the migraine specific analgesic, sumatriptan [33, 42]. The pain hypersensitivity in the periorbit and hind paw produced by NTG injection corresponds to the cutaneous allodynia of the cephalic and extracephalic region in patients with CM [32]. Furthermore, NTG treatment also elicits some migraine-specific symptoms in mice, comprising photophobia, hypoactivity, and facial grimace behaviors, which are similar to the characteristics of photophobia and activities aggravating headache in patients with CM [45, 46]. Migraine is more prevalent in women than in men, and estrogen plays an important role in that sex difference [47, 48]. Numerous studies have shown that the level and stability of estrogen can modulate the transmission of pain pathways [49, 50]. To avoid the interference of estrogen, the male mice were selected to establish the model, since mice of both sexes can be used to successfully establish the NTG-induced CM model [29]. Given the notable sex differences in migraine prevalence, the sex dimorphism of P2X7R involvement in pathogenesis of CM requires further exploration.

Central sensitization is an abnormal state of increased responsiveness of the nociceptive system, which is caused by the enhanced excitability of neurons and circuits in the nociceptive pathway [39]. It is manifested as spontaneous pain hypersensitivity in response to innocuous stimuli (allodynia) and exaggerated as well as prolonged pain in response to noxious stimuli (hyperalgesia) [39]. Extensive studies have revealed the crucial role of central sensitization as the most important pathophysiology of CM [3, 51]. CGRP is a very important neuropeptide involved in the development of CM and has been widely used as the therapeutic target of CM in the clinic [37, 38]. It is synthesized by small diameter sensory neurons in the TG, subsequently released and acts on the postsynaptic CGRPR1 on trigeminal neurons in the TNC and thus participates in the central sensitization [38]. c-fos is the protein encoded by the immediate early gene. Its expression level reflects neuronal activity and has been recognized as a reliable marker mediating central sensitization in pain and other nociceptive stimuli [39, 52]. Accordingly, the expression of CGRP and c-fos were selected to evaluate the central sensitization state, as in our previous studies.

Previous research has explored the pathophysiology of CM, mainly focusing on the neurons in the peripheral nervous system, such as TG. Little attention has been paid to microglia in the CNS. Recently, we found that the activated microglia in the TNC area contribute to central sensitization through releasing the pro-inflammation factors, neurotrophin [8, 10]. Activation of microglial purinoceptors, P2X4R and P2Y12R, and the NLRP3 inflammasome promoted the development and progression of CM [8–10]. Therefore, the investigation of the underlying molecular mechanism in this study mainly focused on the activation of microglia and the inflammatory response. With regard to the microglia-neuron crosstalk underlying central sensitization, our previous works have provided some explanations.

A large number of studies have suggested that the up-regulation of P2X7R is involved in the cancer and neuropathic pain [13, 14]. Here, we reported increase in the expression of microglial P2X7R in the TNC area and confirmed the relationship between P2X7R and CM. Chronic administration of the P2X7R antagonist, BBG, significantly alleviated the mechanical and thermal hyperalgesia induced by recurrent NTG injection. In addition, blocking P2X7R also remarkably reduced the expression of CGRP and c-fos, which were selected to evaluate the central sensitization state. We verified the inhibitory effect of P2X7R on the activation of microglia and the NLRP3 inflammasome in the mice model of CM, which was consistent with the results of other neurological diseases reported previously by other teams [53, 54]. These data indicated that P2X7R blockage may prevent the development of CM and provided a new target for the CM prophylaxis.

With regard to the cellular localization of P2X7R, P2X7R is initially found to be expressed in peripheral hematopoietic cells, lymphocytes, and macrophages [55]. Following in-depth research, it is believed that 67% of the P2X7R is also concentrated in the CNS and mainly expressed in glial cells in the CNS, especially microglia [56, 57]. The presence of P2X7R in neurons has been controversial in the past. However, recent studies have confirmed the expression of P2X7R in neurons, and its activation affects neuronal activity [58]. We observed that P2X7R was mainly expressed in microglia rather than neurons in the TNC. The reasons for this discrepancy may be due to the different experimental animal model and the different experimental tissue. BBG is a specific P2X7R antagonist that can penetrate the blood-brain barrier [16]. It is well known that BBG at micromolar concentrations also inhibits sodium channels in vitro [59]. However, the dose of BBG used in our experiment was considered to be unable to reach an effective concentration to affect the activity of sodium channels in the brain according to a previous study [60]. Additionally, P2X7R gene knockout was reported to completely abolish the analgesic effect of BBG [16]. Therefore, the BBG used in our experiment was specific to P2X7R. Since BBG was administered systematically in our current study, we cannot rule out the potential effect of other pain modulatory regions exemplified by trigeminal ganglion [61], the diencephalic [62], in CM, because P2X7R is also widely expressed in these areas.

Autophagy is a lysosome-dependent degradation process that breaks down intracellular organelles and misfolded protein aggregates to maintain energy homeostasis and control the quality of the proteins and organelles. Abnormal autophagic processes have been implicated in various degenerative diseases, comprising AD, PD, ALS, etc. [63]. Recent evidence has revealed the involvement of autophagy in Nep [27, 28]. It has been reported that the expression of the autophagy-related protein is altered depending on the different models of Nep [64]. In addition, RAPA, a potent autophagy inducer, has been demonstrated to produce a long-lasting analgesic effect on neuropathic pain by inhibiting the activation of microglia, limiting the release of pro-inflammatory factors, and improving nerve myelination [30, 65]. Until now, no research has been conducted to explore the role and specific mechanism of autophagy in migraine. Our current experiment fills this gap.

The LC3-II protein is the most commonly used autophagy marker, and its expression level parallels to the number of autophagosomes [35]. P62 is the endogenous autophagy substrate that recruits the protein for autophagic degradation [35, 36]. The combination of LC3-II and p62 can preliminarily assess the level of autophagic flux [35]. Our data showed that LC3-II was elevated, accompanied by the accumulation of p62 in the NTG-induced CM model, which indicated the blocked autophagic flux featured by suppression of the degradation route. To further confirm the impaired autophagic flux in CM, we used CQ to inhibit the autophagy-lysosome pathway and compared the expression of LC3-II in NTG-treated mice with and without CQ. The results showed that compared with the mice treated with NTG alone, the protein level of LC3-II did not further increase in the mice treated with NTG and CQ, which also supported the impaired autophagic flux in CM. To further determine the role of autophagy in migraine, we administrated the autophagy inducer, RAPA, and the results showed that RAPA attenuated basal rather than acute hyperalgesia and reduced the expression of CGRP and c-fos. These findings supported the preventive function of autophagy activation in CM. In our study, autophagy intervention was achieved through systematic administration of CQ and RAPA, which may also activate potential non-specific effects. To precisely identify the function of autophagy in CM, transgenic mice with autophagic defects are warranted in the future research. In addition, the molecular mechanism underlying autophagy impairment in CM needs to be further explored.

It is well documented that P2X7R plays a dual role in autophagy pathway regulation depending on the different cell types and stimulation window of P2X7R [22]. In non-transgenic microglia and human epithelial cells, P2X7R negatively regulates autophagy by disrupting lysosomal function [20, 66]. Nevertheless, in transgenic microglia, exemplified by SOD1-G93A microglia, dystrophic muscle cells, and monocytes as well as macrophages infected with mycobacteria, P2X7R acts as a positive modulator in autophagy [22]. In addition, short-term stimulation by P2X7R significantly enhances the autophagic flux, while the persistent activation of P2X7R leads to a blockage of autophagic flux [22]. Our present data showed that P2X7R negatively regulated the autophagic flux in CM, which is supported by the reduced expression of LC3-II as well as p62 in response to P2X7R blockage. These results are consistent with those reported by previous studies, since CM is a well-known chronic pain and P2X7R is sustainably activated during the chronification process of pain. We did not explore the exact mechanism of P2X7R regulation of autophagy herein. Some evidence has indicated that p38 MAPK can inhibit autophagy by phosphorylating ULK1 [67, 68]. Meanwhile, the p38 MAPK pathway is the exact downstream of P2X7R in promoting the microglial inflammatory response [69, 70]. Therefore, we speculated that P2X7R might regulate the autophagic process via the p38 MAPK-ULK1 pathway. Certainly, the specific mechanism requires further exploration.

Substantial investigations have reported the complex reciprocal relationship between autophagy and the microglial inflammatory response [71, 72]. However, no studies have examined this mechanism in migraine. It is well documented that autophagic flux can affect the microglial phenotypes and guide the inflammation into a protective or detrimental state [73, 74]. The inhibited autophagic flux has been reported to induce activation of M1 phenotype microglia and promote the release of pro-inflammatory cytokines. While the enhanced autophagy leads to completely reverse results [40, 41]. Autophagy has been shown to decrease the expression of the NLRP3 inflammasome through regulating the mitochondrial damage and degrading the NLRP3 inflammasome [26]. Our present findings are consistent with the involvement of autophagy in the inflammatory response. We demonstrated that abnormal autophagy might contribute to CM pathogenesis by regulating the activation of microglia and subsequent inflammatory response.

## Conclusions

In summary, we found a dysfunctional autophagic flux in the TNC and demonstrated the beneficial effect of autophagy induction against CM progression through inhibiting activation of microglia and production of inflammatory factors. In addition, microglial P2X7R in the TNC was confirmed to participate in the pathogenesis of CM. Blockage of P2X7R alleviated the central sensitization by suppressing microglia and inflammation activation via negatively modulating the autophagic pathway. Our data reveal the modulation of autophagy as a novel mechanism by which P2X7R regulates pain processing. The involvement role of P2X7R and autophagy in CM introduces new information for investigation of the mechanism of CM and provides potential therapeutic targets for CM prevention.

## Data Availability

The data used in this article are available from the corresponding author on reasonable request if necessary.

## Abbreviations

ANOVA: One-way analysis of variance
AD: Alzheimer’s disease
BBG: Brilliant Blue G
CM: Chronic migraine
CGRP: Calcitonin gene-related peptide
CNS: Central nervous system
CQ: Chloroquine
FOV: Field of view
Nep: Neuropathic pain
NTG: Nitroglycerin
OD: Optic density
P2X7R: P2X7 receptor
PVDF: Polyvinylidene difluoride
PFA: Paraformaldehyde
PBS: Phosphate-buffered saline
RAPA: Rapamycin
SDS-PAGE: Sodium dodecyl sulfate-polyacrylamide
SEM: Standard error of the mean
TNC: Trigeminal nucleus caudalis
TBST buffer: Tris-buffered saline with Tween-20
